# Keeping Vegetables in Winter

**Published:** 1882-03

**Authors:** W. D. Philbrick


### KEEPING VEGETABLES IN WINTER.

The storage of the bountiful harvests of autumn, for use in winter, is attracting more and more attention every year, so that our winter market is now almost always well supplied with a variety of roots, such as onions, beets, carrots, turnips, etc., as well as plenty of fresh cabbages, celery, and spinach, and also squashes.

The requirements of these crops for keeping well are very different, and depend upon conditions of temperature and moisture which are easy enough to understand but by no means easy to control in our fickle and ever-changing climate. Thus, the cool, damp air of a cellar is a good place to keep roots and apples, provided they can be kept moist and cold enough to prevent wilting.

This is quite out of the question in the modern dwelling-house cellar, in which the hot-air or steam furnace keeps up a constant disturbance of the air, and produces a dryness and warmth ruinous to preservation of roots. Even when a partition separates the furnace from the store-room, the careless neglect to shut a door or the cracks that often occur in a board partition are frequently the occasion of a dryness and heat in the store room utterly unsuited to the preservation of such products as roots or apples. There are other good reasons for not using the house cellar for storing vegetables; if neglected in spring and summer the remnant that remains at this season frequently becomes a nuisance and a cause of disease in the household. For these reasons it is better to make a cellar under some shed or carriage house, or the barn, for roots and apples.

The conditions required for keeping spinach are very similar, namely, a low temperature, near the freezing point, and very slight circulation of air. A cellar as ordinarily built, mostly under ground, is apt to be too warm, and for this reason a cellar whose walls rise slightly above the surface is better. The spinach is spread upon shelves, with spaces between the boards to allow free circulation of air; usually two or three shelves are built over each other, and the spinach is piled upon them about six inches deep; slight ventilation is needed to keep the spinach well, but not too much, for it is easily damaged by either freezing hard or by wilting; slight freezing does no harm, if it can be kept from thawing frequently. A cold frame covered with sashes and shutters will keep spinach pretty well for a month or two after cutting it, but is too subject to frequent freezing and thawing in changeable weather to answer well for a longer time.

Celery keeps well under similar conditions, except that it should never be allowed to freeze at all after blanching. It is usually stored by heeling it in, quite thickly, in the bottom of a pit, which is covered with boards, and the boards protected from frost by a heavy coat of leaves, spent tan, or eel grass. It will not keep well in a common cellar, unless buried up in sand or loam, the air of a cellar being so dry as to cause it to wilt.

Onions keep well in a cold, dry cellar, if not too early; they should be stored in a dry loft, or on a barn floor until cold weather endangers their freezing, say about Thanksgiving time, when they may be barreled and put in the cellar. They will also keep well on the floor of a loft, if allowed freeze slightly and then covered with hay to prevent frequent and sudden thawing, which injures them; they will endure about as much frost as apples.

To keep other roots, such as beets, carrots, and turnips, from wilting, it is a good plan to put them up in barrels with heads, or to pile them in the cellar about four feet deep, and cover the pile with a little straw or coarse litter, to prevent evaporation. If the cellar is kept cold, they will not sprout and grow; but this is not always easy to do, as mild weather approaches in spring, at which season a good pit keeps the roots in better order than any cellar can do.

To keep squashes well very different conditions are essential. The squash is a tropical plant and will not well endure cold weather; even an approach to the freezing point below 40° injures them for keeping, and if the temperature can be kept uniformly above 50° from the time they are harvested, it will be all the better; free circulation of air is essential also, especially when they are first gathered, and for several weeks afterward. A cellar with a furnace in it, where the temperature is above 50°, will keep them well. But a cellar without a fire is too damp and cold, and they will not keep long in it.

One of the best places to be found in most houses for keeping squashes is a closet against the kitchen chimney. They need looking over every two weeks to pick out the specked ones; they keep pretty well till spring, if carefully watched; the hard-shelled squashes, like the Hubbard end Marblehead, keep very much better than the turban and marrow varieties, which are mostly used in autumn for pies, etc.

Tomatoes picked quite green, just before frost endangers their destruction, and spread out upon the benches of a greenhouse or under the glass of a hot bed, will ripen after several days' exposure to the warmth of the sun, and prove very acceptable after the frost has destroyed the vines.

String beans may be easily dried by spreading them on a roof or other convenient place, and furnish an excellent winter dish; they need only to be soaked as if freshly picked. This method is not so generally known and practiced as it should be. String beans are a delicate dish in midwinter and well worth the slight trouble of saving them. Lima beans, shelled and dried, make most excellent stewed beans in the winter season, so much better than ordinary pea beans that one would be quite surprised at the difference who had never tried it.

The method of preserving cabbages for winter will be described in another paper.—*W. D. Philbrick, N. E. Farmer.*

